# Gender differences in symptom interactions between problematic smartphone use and social anxiety in adolescents: a network analysis

**DOI:** 10.1186/s13034-025-00865-w

**Published:** 2025-02-14

**Authors:** Sipu Guo, Xinyuan Zou, Yanqiang Tao, Yichao Lv, Xiangping Liu, Silin Huang

**Affiliations:** 1 Institute of Developmental Psychology, Faculty of Psychology, Beijing Normal University, 19, Xinjiekouwai Street, Beijing, 100875 China; 2 Beijing Key Laboratory of Applied Experimental Psychology, National Demonstration Center for Experimental Psychology Education, Beijing, 100875 China

**Keywords:** Problematic smartphone use, Social anxiety, Gender difference, Adolescent, Network analysis

## Abstract

**Background:**

Increased prevalence of problematic smartphone use (PSU) in adolescents, results in a cycle of interaction between PSU and social anxiety. However, it is still unknown whether PSU and social anxiety symptoms have interacted among adolescents and whether there are gender differences in these symptoms. Therefore, this study investigated the gender differences in the symptom interactions between PSU and social anxiety via symptom network analysis.

**Methods:**

This study included 2918 adolescents (52.71% boys; *M*_age_ = 14.73, *SD*_age_ = 1.39) from junior and senior high schools in China. The Mobile Phone Addiction Index and Social Anxiety Scale were used to evaluate symptomatology and networks. Network analysis and network comparison tests were used to determine the network structure, centrality, bridge symptoms and gender differences in the PSU-social anxiety network among adolescents.

**Results:**

The most influential symptoms were “productivity loss” and “afraid of negative evaluation”. “Afraid of negative evaluation” was the bridge through which PSU was related to social anxiety. Gender differences were not found in network strength but occurred in network structure. Although girls reported more social anxiety, boys had a tighter network structure. The correlation between PSU and social anxiety was greater in boys than in girls. The “inability to control craving” was particularly critical for girls, while “feeling anxious and lost” was prominent for boys.

**Conclusions:**

The current study highlights the symptom interactions between PSU and social anxiety among adolescents and the gender differences in network structures. Further intervention that targets “afraid of negative evaluation” may disassociate the interaction between PSU and social anxiety symptoms. In particular, changing girls’ cognitive ability (e.g., inhibition) and boys’ negative emotions are potentially effective means of intervention. The limitations of the cross-sectional design and data-driven methodology necessitate interpreting the results with caution.

**Supplementary Information:**

The online version contains supplementary material available at 10.1186/s13034-025-00865-w.

## Introduction

Problematic smartphone use (PSU) is the excessive and compulsive use of smartphones and affects users’ daily lives (e.g., productivity, social relationships, or mental health) [1]. About 90% of Chinese adolescents use smartphones daily, and PSU is a critical issue [2]. Approximately 16% of adolescents exhibit PSU [3], and among those referred to psychiatry clinics, this figure rises to 50.6%, often accompanied by psychiatric symptoms such as depression, anxiety, and interpersonal sensitivity [4]. In other words, there may be a close relationship between PSU and mental health.

The relationship between PSU and psychiatric symptoms is complex. Among the many psychological problems associated with PSU, social anxiety is of greater concern to adolescents. Social anxiety are common in adolescents and is even referred to as “disorders of adolescence” [5]. Those worsening symptoms are associated with psychiatric comorbidities [6]. We believe that focusing on the interaction between symptoms of different disorders may help explain why comorbidities are associated with more severe symptoms and find a more targeted way to alleviate psychological problems. Therefore, by adopting network analysis, this study aimed to explore the key interactions of adolescents’ PSU and social anxiety symptoms, as well as gender differences in these interactions.

### The interactions between PSU and social anxiety symptoms in adolescents

Individuals with social anxiety are generally described as avoiding social activities despite the desire to relate to others, fearing that they are not as popular, intelligent, or interesting as they would like [7]. Previous studies have focused mainly on the prediction of social anxiety on adolescents’ PSU. When people want to gain social appreciation or avoid social criticism, they are more inclined to use their smartphones to seek more and closer interpersonal relationships [8]. Adolescence is a sensitive period of neural development, and adolescents are especially sensitive to social connectedness and loneliness [9]. Therefore, adolescence is considered a period with a high prevalence of social anxiety. In China, amidst rapid social change over the past two decades (2002 to 2020), there has been a notable increase in social anxiety levels among Chinese adolescents [10], who appear to experience higher social anxiety than Western peers, with no difference in interpretation bias [11]. Additionally, social anxiety may contribute to some addictive behaviors, such as substance use [12] or PSU [13].

Chinese adolescents experience substantial pressure from traditional cultural values and intense societal competition [14]. Based on the compensatory internet use theory (CIUT) [15], PSU is thought to be an excessive behavior that reduces the negative emotions associated with stressful events (e.g., academic demands and parental expectations). Ideally, adolescents should choose more adaptive behaviors, such as finding social support and developing positive social relationships. However, considering the ubiquity of smartphones and their social attributes, using a smartphone often becomes the first choice for adolescents. In particular, a majority of previous studies found social anxiety to be a significant predictor of PSU [16, 17]. Similar results were found in a review study of social anxiety symptoms associated with PSU [18].

However, PSU may lead to social anxiety. For example, some studies have shown that PSU results in social anxiety in adolescents [19, 20]. Based on the cognitive-behavioral model of psychopathology [21], PSU produces false socialization that tends to cause people to acquire social anxiety. Therefore, social anxiety and PSU may have a bidirectional relationship [22]. These findings suggested that PSU and social anxiety interact and influence each other. Moreover, this bidirectional relationship may hint at a possible vicious cycle between social anxiety and PSU.

Most previous studies have focused on the more macroscopic relationship between social anxiety and PSU, especially the unidirectional relationship. However, focusing solely on the relations between disorders neglects the interactions, development, and heterogeneity of symptoms. To alleviate the vicious cycle, exploring the complex interaction relationship between PSU and social anxiety symptoms is particularly important. From a network perspective, the interaction between symptoms forms a psychological disorder, and further cycles promote this formation and maintenance of the disorder [23]. Network analysis can reveal the most influential factors (i.e., central symptoms, which are most associated with other symptoms within the disorder) that reflect the interconnections between symptoms.

Furthermore, identifying bridge symptoms and centrality can effectively improve the research on and practice of identifying mental disorder comorbidities [24]. Borsboom [23] indicated that a bridge symptom is a symptom from disorder A that is linked to symptoms from disorder B. This connection shows that the two disorders can happen together in a person, creating a pattern called comorbidity. Therefore, this study used network analysis to explore whether and how social anxiety and PSU interact on symptoms, that is, specific central symptoms and bridge symptoms.

### Gender differences in the interaction of PSU and social anxiety symptoms

Although several studies have investigated gender differences in PSU and social anxiety, it remains a confusing topic. On the one hand, some studies have shown no gender differences in PSU or social anxiety symptoms. For example, a study revealed the unfeasibility of predicting social anxiety severity through gender [25]. There is no significant difference between men and women in terms of making mobile phone calls or addiction to mobile games [26]. On the other hand, multiple studies have shown that gender is associated with differences in psychological characteristics and the probability of addiction in PSU. Girls than boys are diagnosed with social anxiety [12] and PSU [27]. Females exhibited significantly higher PSU, with greater frequency and duration [28], potentially linked to heightened social stress and increased social smartphone use [29]. Tending to use mobile phones contributes to poor mental health, especially in females [30]. Other studies show that boys may exhibit a greater tendency to engage with non-social, gadget-oriented functionalities of mobile phones [31]. Males who are addicted to mobile phones are more likely to suffer from anxiety [32].

The current state of social anxiety and PSU research provides an incomplete picture of differences in their relationship for boys and girls. Empirical studies on gender differences in social anxiety and PSU have focused mainly on how gender moderates the relationship between social anxiety and PSU. Few studies have examined the gender difference in the comorbidity of social anxiety and PSU. Network analysis can provide insights into the interactions of social anxiety and PSU symptoms and the gender differences in these symptoms. Network analysis can uncover gender differences in social anxiety and PSU by comparing network structure (i.e., how symptoms are connected) and global strength (i.e., the sum of the strengths of all edges in the network). Therefore, this study aimed to explore whether and how network structures, core symptoms, and bridge symptoms of social anxiety and PSU differ between boys and girls based on a large sample size.

### The current study

The interaction of social anxiety and PSU symptoms among adolescents and the gender differences in these symptoms remain unclear. Methodologically, the lack of symptom structures regarding the complex relationships among gender, social anxiety, and PSU in previous studies leaves multiple explanations open. Therefore, the network approach offers a novel approach to unpacking this problem [33].

In the current study, we adopted network analysis to explore the gender differences in the network structure centrality and bridge symptoms of social anxiety and PSU among adolescents. This study aimed to examine two hypotheses: (1) interactions between PSU and social anxiety symptoms occur among adolescents, and (2) there are gender differences in the central symptoms and bridge centrality indices of the social anxiety-PSU-related symptomatology network among adolescents.

## Method

### Participants

A total of 3083 adolescents from several public (junior and senior high) schools across county and city in Shanxi Province, China, from June to July 2023 using Wenjuanxing (an online questionnaire software; https://www.wjx.cn). No students included in the study presented medical records indicating diagnoses of major depressive disorder, generalized anxiety disorder, schizophrenia, or critical physical illnesses. Among them, 165 participants were excluded for failing the attention check (i.e., “Please select C from A, B, and C.”). The final valid dataset included 2918 participants (*M*_age_ = 14.73, *SD*_age_ = 1.39; 52.71% boys). Other demographic information (e.g., school location, left-behind children, only child, health problems, parental marriage) is presented in Table S1.

All the questionnaires were distributed and collected by teachers who were instructed and trained. All the participating students and parents were informed of the purpose of this study and signed electronic informed consent forms. The participants agreed to participate in the study and were given the option to withdraw from the study at any time. The present study was approved by the Institutional Review Board of the authors’ affiliated institution (Reference number: 202305150086).

### Measures

#### Mobile phone addiction index (MPAI)

Problematic smartphone use was evaluated using the mobile phone addiction index (MPAI) developed by Leung [34]. This self-report questionnaire consists of 17 items (e.g., “*You can never spend enough time on your mobile phone*”) rated on a 5-point Likert scale with 1 = “not at all,” 2 = “rarely,” 3 = “occasionally,” 4 = “often,” and 5 = “always.” The MPAI comprises four factorial components: inability to control craving (7 items), feeling anxious and lost (5 items), withdrawal or escape (3 items), and productivity loss (2 items). The average score of all 17 items was calculated, and higher MPAI scores are indicative of higher levels of PSU. In this study, the Cronbach’s *α* value for the MPAI was 0.95, reflecting high internal consistency.

#### Social anxiety scale (SAS)

The social anxiety scale was developed initially by La Greca and Lopez [35]. The Chinese version of the social anxiety scale consists of 13 items encompassing three dimensions: afraid of negative evaluation (8 items), anxiety in unfamiliar situation (6 items), and anxiety in normal situation (4 items). Each item (e.g., “*I'm always worried that people won't like me*”) is rated on a 5-point Likert scale, labeled as “is true for you”, with responses ranging from 1 (not at all) to 5 (all the time). The average score of all 13 items was calculated, with higher scores representing higher levels of social anxiety. The social anxiety scale exhibited high internal consistency in this study, with a Cronbach’s *α* value of 0.97.

### Statistical analysis

All analyses were performed in version 4.3.1 of R software [36]. Descriptive statistics were performed on the overall sample, and a preliminary exploratory analysis of the differences between the boys and girls on both scales (social anxiety and PSU) was conducted.

The R package *qgraph* visualizes the correlation matrix by representing symptoms as nodes and partial correlations as edges, with edge width reflecting the magnitude of the correlation while controlling for the effects of other variables [37]. The graphical least absolute shrinkage and selection operator (LASSO) method enhances graph sparsity and diminishes the probability of false positives being incorporated as edges [38]. To determine the optimal network structure, the extended Bayesian Information Criterion (EBIC) was employed [38]. Node centralities, measured by Expected Influence (*EI*), quantify a node's importance in a network by summing the strengths of all its connections, including both positive and negative edges. Bridge symptoms (i.e., bridge *EI*) were discriminated against using the R package *networktools* [39] to investigate potential key symptoms that connect social anxiety and PSU. When calculating a node's bridge *EI*, only its connections to nodes in other disorders are included, while connections within its own disorder community are ignored. In addition, the R package *mgm* was used to calculate predictability, which shows how well a node can be predicted by other neighboring nodes [40]. In the resulting undirected network (i.e., the non-causal nature of network), nodes represent PSU and social anxiety variables, while edges reflect pairwise regularized partial correlations between these variables.

The present study employed a bootstrapping approach, implemented using the R package *bootnet* 1.4.3 [38], to minimize the potential bias in network results stemming from self-reported data and data-driven methodologies and enhance the robustness of the findings. Firstly, we applied the nonparametric bootstrap method to calculate 95% confidence intervals (CIs) for the accuracy of edge weights by resampling the data 1000 times (with replacement), thereby establishing the distribution of edge weights. Subsequently, bootstrapped difference tests were conducted to evaluate differences in network properties (i.e., edge weights and node strengths), minimizing the potential bias associated with data-driven methodologies. Secondly, a case-dropping bootstrap procedure, by sampling the data 1000 times (with replacement), was used to assess the stability of centrality indices, resulting in the correlation stability coefficient (*CS-C*), minimizing the potential bias associated with self-reported data. Typically, *CS-C* values ≥ 0.25 indicate satisfactory stability.

To test the gender differences in the social anxiety-PSU network, we used the R package *NetworkComparisonTest* (NCT) [41] for further analysis. Specifically, a total of four tests were used, including a network structure invariance test (i.e., the difference in the maximum edge strength of networks), a global strength invariance test (i.e., the difference in summation of edge strengths), and an edge strength invariance test (i.e., differences between certain edges in a network). In addition, the centrality invariance test was used to compare the difference in the *EI* of nodes between boys and girls.

To avoid sociocultural factors that may influence the interpretation of network results, we performed a sensitivity analysis [42], including demographic characteristics between genders as covariates, to control for potential confounding effects. This study computes the weighted adjacency matrices of networks both with and without demographic characteristics, considering the significant demographic differences between genders. The correlation between the two matrices, which include symptoms of PSU use and social anxiety, is then calculated. If the correlation is high, it suggests that the significant demographic differences between genders have a negligible effect on the results, indicating that the main findings are robust and not substantially influenced by these factors.

## Results

### Descriptive statistics and symptom

**Fig. 1:**
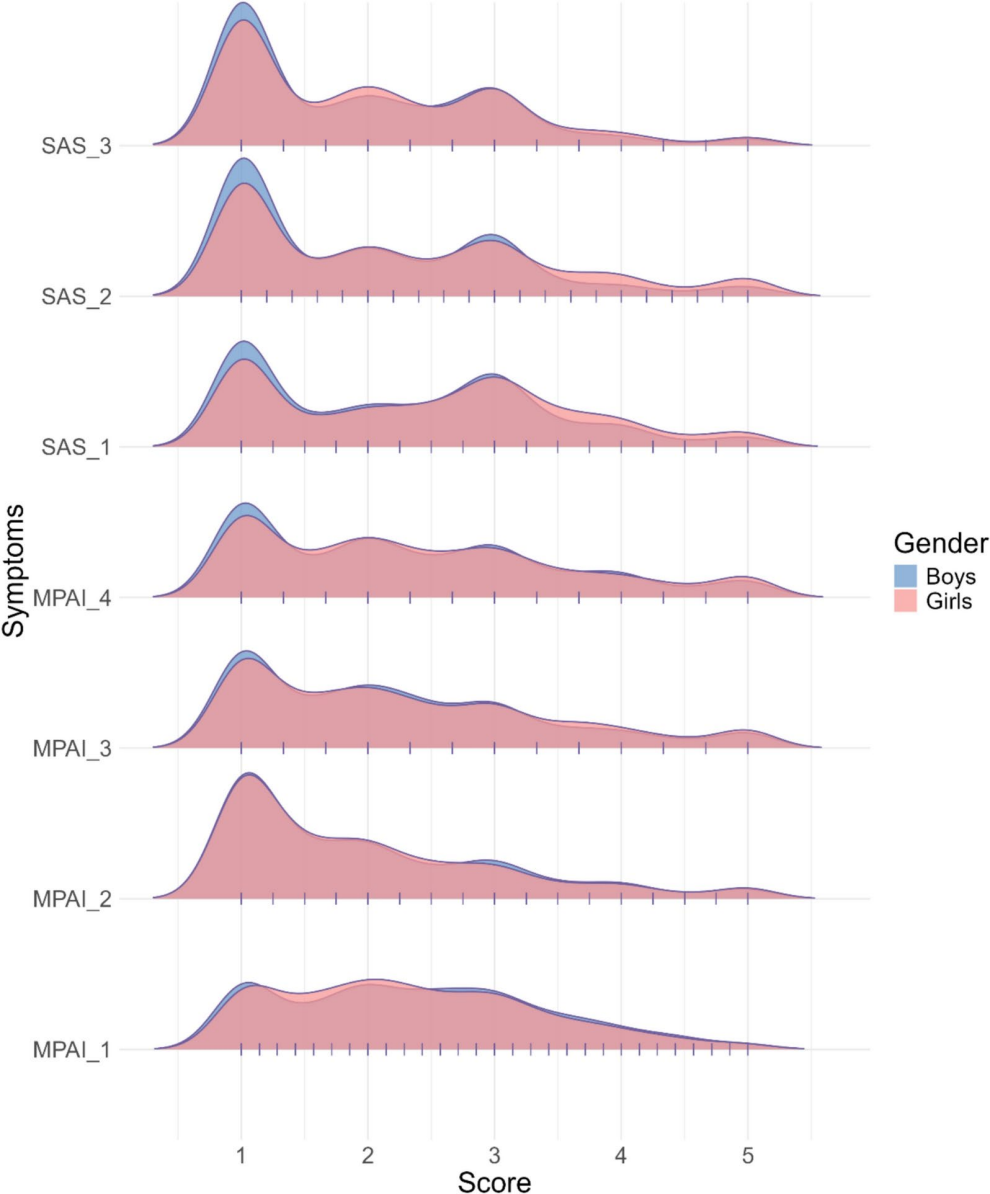
Density of symptom scores between genders. Pink represents the density of girls' scores on each dimension and blue represents the density of boys' scores on each dimension

Density of symptom scores across genders are shown in Fig. 1. The means and standard deviations are shown in Table 1. Specifically, there were no significant gender differences in the four dimensions of PSU (*p* > 0.05). However, in the case of social anxiety, girls scored significantly higher than boys on all three dimensions (*p* < 0.05). The demographic characteristics are presented in Table S1, which shows significant gender differences in education level, being an only child, parental relationships, parental divorce, and the parent with whom individuals reside after a divorce (*p* < 0.05).

**Table 1:** Description of statistics and gender differences in problematic smartphone use and social anxiety

Node (Dimension)	All participants		Boys		Girls		Statistics (t)	Cohen’s *d*
	*M*	*SD*	*M*	*SD*	*M*	*SD*		
Problematic smartphone use								
MPAI 1: Inability to control craving	2.37	1.01	2.39	1.03	2.35	1.00	1.05	0.03
MPAI 2: Feeling anxious and lost	1.98	1.07	1.99	1.07	1.98	1.07	0.43	0.00
MPAI 3: Withdrawal/escape	2.25	1.15	2.21	1.13	2.29	1.17	− 1.58	− 0.07
MPAI 4: Productivity loss	2.36	1.19	2.32	1.18	2.40	1.19	− 1.68	− 0.07
Social anxiety								
SAS 1: Anxiety in unfamiliar situation	2.36	1.15	2.25	1.11	2.45	1.17	− 4.55***	− 0.18
SAS 2: Afraid of negative evaluation	2.12	1.15	2.00	1.08	2.24	1.20	− 5.55***	− 0.21
SAS 3: Anxiety in normal situation	1.97	1.03	1.92	1.02	2.02	1.04	− 2.36**	− 0.09

*M*: mean; *SD*: standard deviation; MPAI: Mobile Phone Addiction Index; SAS: Social Anxiety Scale

^**^p < 0.05

^***^p < 0.01

Symptom check was conducted by calculating symptom informativeness (measured by the standard deviation of the symptom) and symptom redundancy. Results found that no symptom lacked informativeness (i.e., no symptom had a standard deviation more than 2.5 SD below the mean informativeness level), and no symptom showed redundancy with any other symptom (i.e., fewer than 25% of the correlations were significantly different).

### The network of PSU and social anxiety symptoms among all adolescents

#### Network structures

**Fig. 2:**
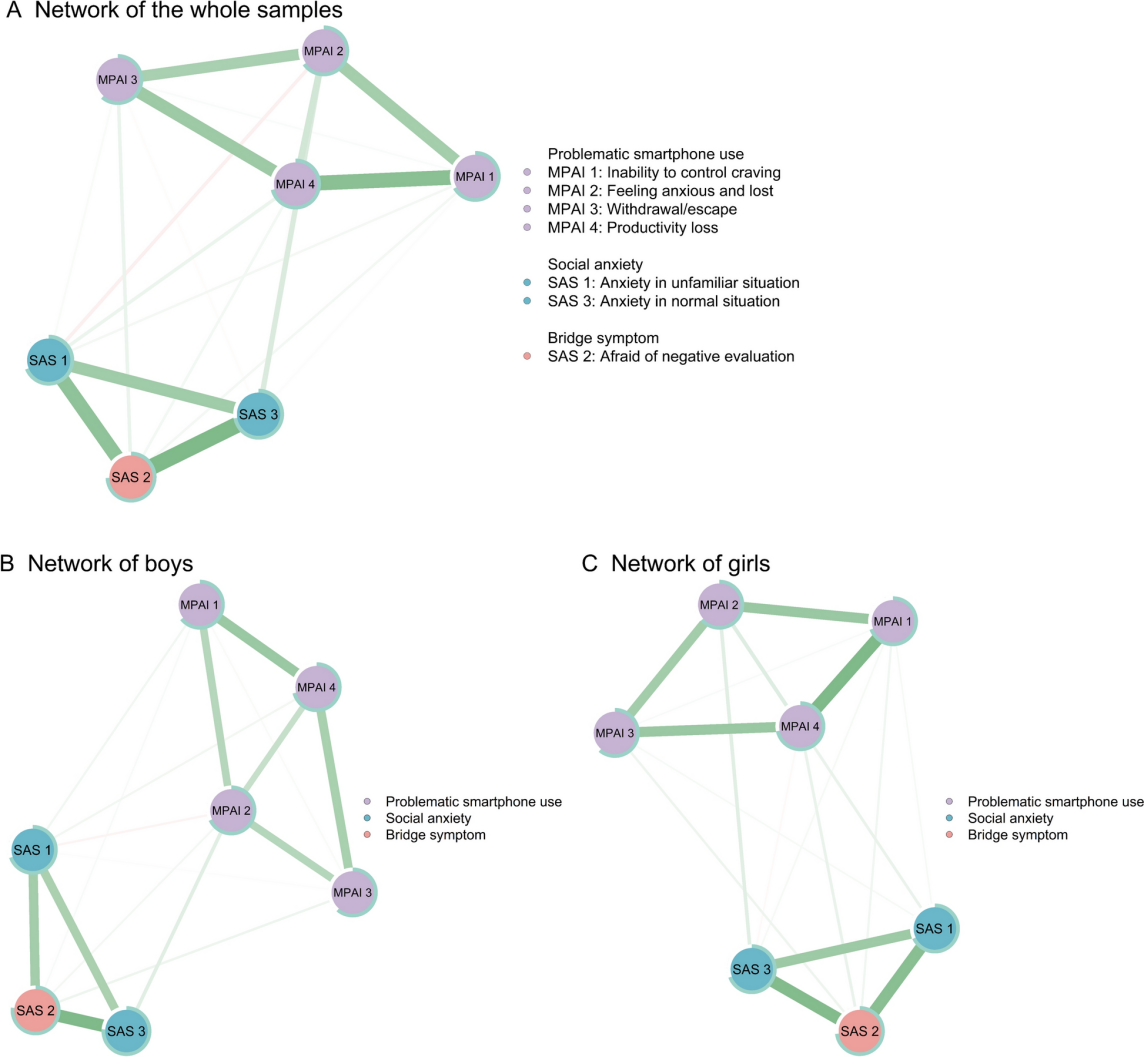
Network structures and bridge symptoms of PSU and social anxiety symptoms in adolescents. The green color of the edges indicates a positive correlation, and red indicates a negative correlation. **A** Network of the whole samples. **B** Network of boys. **C** Network of girls

The full networks, consisting of 7 nodes and 21 edges (7*(7–1)/2), are shown in part A of Fig. 2, further details are provided in Table S2. As shown in Fig. 2, there is a strong intrinsic connection between the dimensions, with each of the four dimensions in PSU and each of the three dimensions in social anxiety tending to cluster together.

The network (see part A of Fig. 2) contains 20 (95.24%) nonzero weight edges, consisting of 17 positive edges and 3 negative edges. This finding suggests that severity of most symptoms in this network increase with the severity of other symptoms. Among these correlations, the three most prominent positively correlated relations between PSU and social anxiety were #MPAI 2 (“feeling anxious and lost”) and #SAS 3 (“anxiety in normal situation”) (*r* = 0.13); #MPAI 3 (“withdrawal/escape”) and #SAS 2 (“afraid of negative evaluation”) (*r* = 0.08); and #MPAI 4 (“productivity loss”) and #SAS 2 (“afraid of negative evaluation”) (*r* = 0.04). The results of the edge-weight difference test (part A of Figure S1) suggest that these differences were significant. Moreover, the nonparametric bootstrap data indicated that the 95% confidence intervals were narrow for all adolescent networks (part A of Figure S3), suggesting that the entire adolescent network was accurate and stable.

#### Centrality and bridge symptoms

**Fig. 3:**
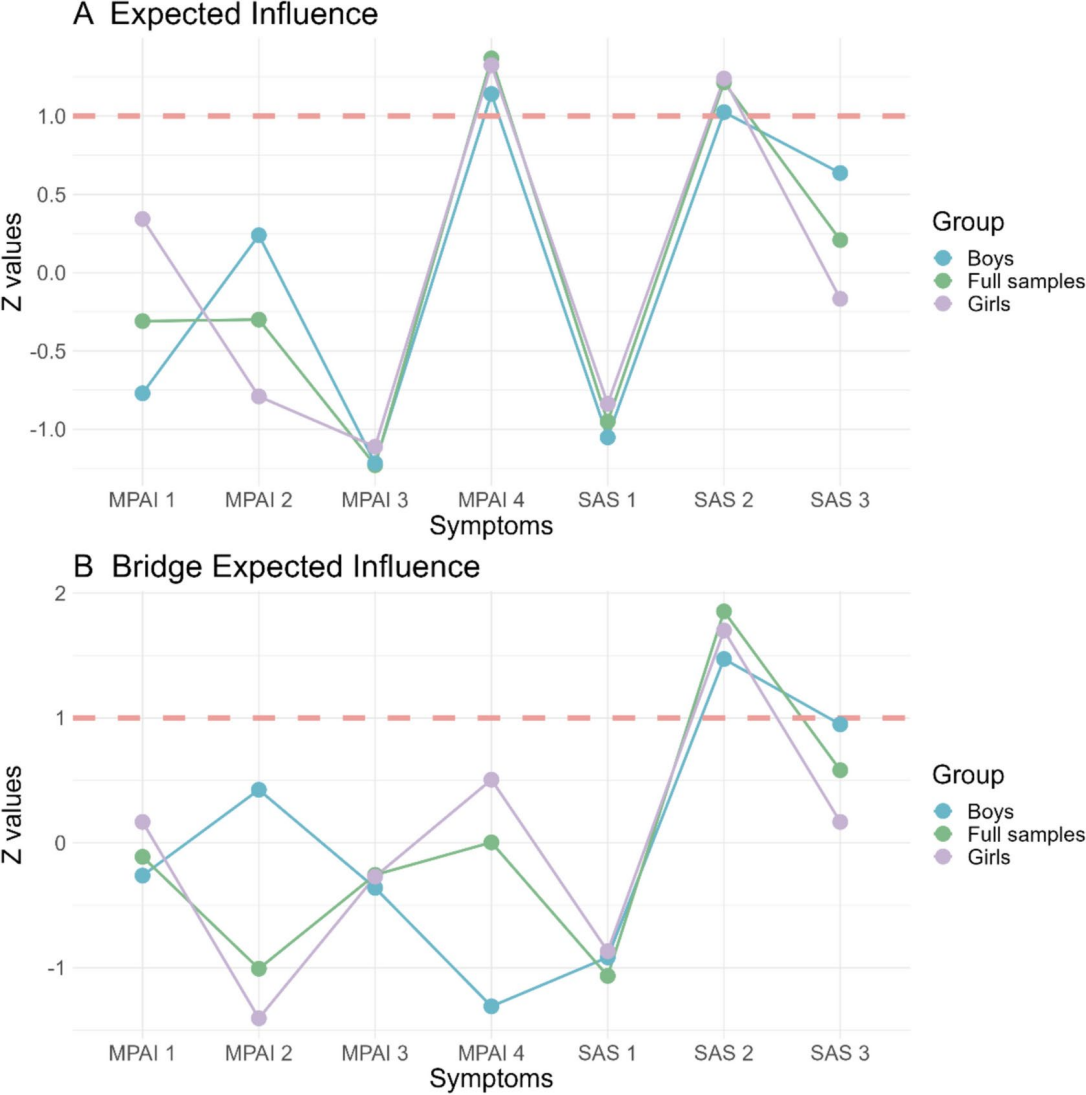
Centrality, and bridge symptoms. **A** Centrality symptoms value (i.e., expected influence; *EI*). **B** Bridge symptoms values (i.e., bridge expected influence; *BEI*). Note that the red dotted line represents the *EI* of 1 (standardized)

For the centrality index (see part A of Fig. 3), #MPAI 4 (“productivity loss”; *EI* = 1.38), #SAS 2 (“afraid of negative evaluation”; *EI* = 1.25), and #SAS 3 (“anxiety in normal situation”; *EI* = 0.16) were identified as the nodes with highly effective influence.

**Fig. 4:**
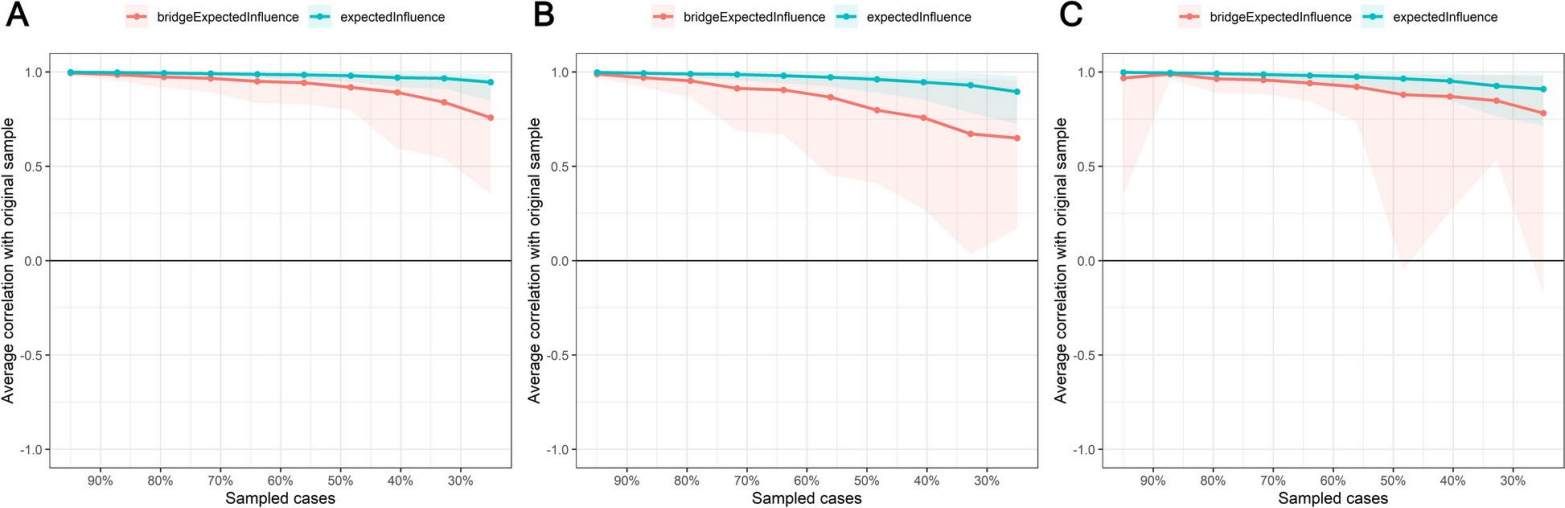
The x-axis represents the percentage of cases from the original sample included at each step. The y-axis represents the average correlations between the original network’s centrality indices and those obtained from the networks re-estimated after excluding increasing percentages of cases. **A** Full samples. **B** Boys. **C** Girls

For the bridge symptom, #SAS 2 (“afraid of negative evaluation”; *EI* = 1.85) was identified as the only bridging symptom among the total sample (see part B of Fig. 3), which means intervention that targets “afraid of negative evaluation” may disassociate the interaction between PSU and social anxiety symptoms. The predictability of #SAS 2 (“afraid of negative evaluation”; 74%; see Table S2) was the highest network structure in all adolescents. The strongest *EI* and bridge *EI* exhibited statistically significant differences from other nodes (part A of Figure S2). This shows that the *EI* of node #SAS 2 (“afraid of negative evaluation”) is more efficient and stable than that of the other nodes. The high-level *EI* (*CS-C* = 0.75) and bridge *EI* (*CS-C* = 0.60; see part A of Fig. 4) suggest that the network stability of the adolescents was excellent.

### *Gender* differences in the network of PSU and social anxiety symptoms

#### Network structure, centrality, and bridge symptoms

The number of nodes and edges in the networks of boys and girls is equal to the number obtained from the overall sample (both 7 nodes and 21 edges; see parts B and C of Fig. 2). The boy network (see Part B of Fig. 2) consists of 17 (80.95%) nonzero-weight edges, including 16 positive edges and only one negative edge. In the boy network, there was a high positive correlation across symptoms of social anxiety. The strongest associations (the top three) between PSU and social anxiety were #MPAI 2 (“feeling anxious and lost”) and #SAS 3 (“anxiety in normal situation”) (*r* = 0.12); #MPAI 3 (“withdrawal/escape”) and #SAS 2 (“afraid of negative evaluation”) (*r* = 0.07); and #MPAI 1 (“inability to control craving”) and #SAS 1 (“anxiety in unfamiliar situation”) (*r* = 0.06).

The girl network (see Part C of Fig. 2) consists of 19 (90.48%) nonzero-weight edges, including 17 positive and two negative edges. The three most notable correlations between PSU and social anxiety were #MPAI 2 (“feeling anxious and lost”) and #SAS 3 (“anxiety in normal situation”) (*r* = 0.10); #MPAI 4 (“productivity loss”) and #SAS 2 (“afraid of negative evaluation”) (*r* = 0.08); and #MPAI 4 (“productivity loss”) and #SAS 1 (“anxiety in unfamiliar situation”) (*r* = 0.08). Therefore, in the girls’ network, strong positive correlations were mainly present for both PSU and social anxiety symptoms. The results of the edge-weight difference test can be found in parts B and C of Figure S1. Moreover, the nonparametric bootstrapped analyses showed that the 95% confidence intervals were narrow for the networks of the boy and girl samples, indicating high stability for each network (see parts B and C of Figure S3).

For the centrality index among boys (see part A of Fig. 3; Table S2), #MPAI 4 (“productivity loss”) (*EI* = 1.12), #SAS 2 (“afraid of negative evaluation”) (*EI* = 1.09), and #SAS 3 (“anxiety in normal situation”) (*EI* = 0.56) were identified as the nodes with the highest effective influence. In comparison, for girls, #MPAI 4 (“productivity loss”) (*EI* = 1.39) had the highest effective influence, followed by #SAS 2 (“afraid of negative evaluation”) (*EI* = 1.28) and #MPAI 1 (“inability to control craving”) (*EI* = 0.18). Generally, #SAS 2 (“afraid of negative evaluation”) was identified as the only bridge symptom among both boys and girls (see part B of Fig. 3). The predictability of the #SAS 2 (“afraid of negative evaluation”; boys = 77%, girls = 73%; Table S2) was highest for both the boy and girl network structures. To test network accuracy and stability, corresponding case-dropping subset sample bootstrap procedures were also conducted and showed that the *EI*s (both *CS-Cs* = 0.75) and bridge *EI*s (boys *CS-C* = 0.36, girls *CS-C* = 0.51; see parts B and C of Fig. 4) were highly stable for boys and girls. The results of the node *EI*s and bridge *EI*s difference test can be found in parts B and C of Figure S2.

#### Network comparison between boys and girls

The network invariance test revealed significant gender differences in the network structures (*M* = 0.12, *p* = 0.044), suggesting that the overall structures were different across boys and girls. The maximum edge [#MPAI 2 (“feeling anxious and lost”)−#MPAI 4 (“productivity loss”)] strength of the boy network (weight = 0.23) was greater than that of the girl network (weight = 0.11). The connection between #MPAI 2 (“feeling anxious and lost”) and #MPAI 4 (“productivity loss”) was reinforced in boys. However, the global strength invariance test showed that the network strength did not significantly differ (diff = 0.07, *p* = 0.626) between boys (global strength = 3.34) and girls (global strength = 3.41).

The edge invariance test showed significant differences in edge weights of the three edges between boys and girls (*p* < 0.05; Table 2). Specifically, #MPAI 2 (“feeling anxious and lost”) was more strongly connected with #MPAI 4 (“productivity loss”) (*p* = 0.010) and #SAS 2 (“afraid of negative evaluation”) (*p* = 0.024) in boys. Boys demonstrated a stronger connection between #SAS 2 (“afraid of negative evaluation”) and #SAS 3 (“anxiety in normal situation”) (*p* = 0.029) than girls. The edges of the PSU-social anxiety network were more tightly connected in boys than in girls.

**Table 2:** The results of edge invariance test between boys and girls

Node 1	Node 2	*p*
MAPI 1	MAPI 2	0.115
MAPI 1	MAPI 3	0.975
MAPI 2	MAPI 3	0.630
MAPI 1	MAPI 4	0.239
MAPI 2	MAPI 4	0.010
MAPI 3	MAPI 4	0.924
MAPI 1	SAS 1	0.466
MAPI 2	SAS 1	0.697
MAPI 3	SAS 1	0.755
MAPI 4	SAS 1	0.538
MAPI 1	SAS 2	0.554
MAPI 2	SAS 2	0.024
MAPI 3	SAS 2	0.857
MAPI 4	SAS 2	0.083
SAS 1	SAS 2	0.306
MAPI 1	SAS 3	0.347
MAPI 2	SAS 3	0.666
MAPI 3	SAS 3	1.000
MAPI 4	SAS 3	0.476
SAS 1	SAS 3	0.982
SAS 2	SAS 3	0.029

Notably, the centrality invariance test showed significant gender differences. The *EI* of #MPAI 1 (inability to control craving) was significantly greater for girls (0.18) than for boys (− 0.71; *p* = 0.008). The *EI* of #MPAI 2 (feeling anxious and lost) and # SAS 3 (“anxiety in normal situation”) was significantly greater for boys (0.25; 0.56) than for girls (-0.78, *p* = 0.004; − 0.22, *p* = 0.019). In the presence of PSU and social anxiety, #MPAI 1 (“inability to control craving”) was more severe in girls than in boys, and in turn, #MPAI 2 (“feeling anxious and lost”) and #SAS 3 (“anxiety in normal situation”) were more severe in boys than in girls.

#### Sensitivity analysis

For the entire sample, sensitivity analysis revealed a high correlation between the seven primary variable matrices in the covariate network matrix and the initial network matrix (i.e., the network without covariates,* r* = 0.986, *p* < 0.001). Moreover, we estimated networks for both boys and girls, incorporating certain demographic characteristics as covariates. The results showed a high correlation between the seven primary variable matrices in the covariate network matrix and the non-covariate network matrix for both groups (boys: *r* = 0.996, p < 0.001; girls: *r* = 0.994, *p* < 0.001). Therefore, gender differences in demographic characteristics (i.e., education level, being an only child, parental relationships, parental divorce, and the parent with whom individuals reside after a divorce) did not affect the network estimation.

## Discussion

The current study used network analysis to explore the interaction between PSU and social anxiety symptoms among adolescents and the gender differences in these symptoms. From the network perspective, the findings of this study revealed core symptoms in the total sample of adolescents and in boys and girls. #SAS 2 (“afraid of negative evaluation”) was identified as the bridging symptom associated with the interaction between PSU and social anxiety. Moreover, gender differences were not found in network strength but were found in network structure.

### Core symptoms of social anxiety and PSU among all participants

Our study revealed that #SAS 2 (“afraid of negative evaluation”) and #MPAI 4 (“productivity loss”) are common and influential symptoms among adolescents. First, #SAS 2 (“afraid of negative evaluation”) involves concerns about being criticized, rejected, and embarrassed and has been recognized as resulting from cognitive bias in those with social anxiety [43]. Developing a symptom of social anxiety with a high expected influence (i.e., #SAS 2: “afraid of negative evaluation”) may pose a greater risk than one with low expected influence but a high absolute score (e.g., #SAS 1: “anxiety in unfamiliar situations”), as the former is more likely to activate additional nodes. These findings echo those of a previous study in which “afraid of negative feelings” served as a hallmark cognitive feature of social anxiety among adolescents and may lead to increased negative emotions (e.g., sadness) and decreased participation in social activities [44].

Second, for “productivity loss”, spending too much time on a smartphone leaves things (e.g., academic work) that should be done unfinished. Using smartphones may prevent adolescents from completing their academic work, which may manifest as “productivity loss” and affect their academic performance in the long term [45]. Academic achievement holds particular significance within the Chinese context. According to a previous study, the self-evaluation of Chinese adolescents is closely related to their academic performance [46], highlighting the broader impact of productivity loss. Moreover, #SAS 2 (“fear of negative evaluation”) and #MPAI 4 (“productivity loss”) showed the third strongest associations between PSU and social anxiety, potentially elucidating their close relationship. If this network model is applicable to longitudinal data, productivity loss could lead individuals to develop a fear of negative evaluation in social contexts. Reduced academic performance associated with “productivity loss” may be linked to increased concerns about negative evaluation, which could, in turn, be associated with heightened symptoms of social anxiety. It is important to note that networks based on cross-sectional data do not establish causality, but rather generate hypotheses regarding causality. Therefore, the interaction described in this study represents a potential hypothesis of causality.

### “Afraid of negative evaluation” is the key bridge symptom in the interactions between social anxiety and PSU networks

#SAS 2 (“afraid of negative evaluation”) was identified as a bridge symptom in all adolescents, consistent with centrality results indicating its significant role in maintaining both social anxiety and PSU. #SAS 2 (“afraid of negative evaluation”) was more influenced by other symptoms (i.e., high predictability value), highlighting its status as a hallmark symptom in the interaction between social anxiety and PSU. When people are frustrated by unmet psychological needs and suffer from negative feelings such as sadness and loneliness, they tend to use mobile phones excessively to escape from these uncomfortable feelings and compensate for their unfilled mental needs [47]. Adolescent smartphone use is more likely to be influenced by peer social relationships [48]. Hence, focusing on "afraid of negative evaluations" may enhance understanding of the interaction between social anxiety and PSU.

Intervention to alleviate symptoms is one of the most effective ways to alleviate the comorbidity of social anxiety and PSU [24]. #SAS 2 (“afraid of negative evaluation”) is an important domain-level symptom in the initiation and/or maintenance of comorbidity for these syndromes, higher dimensions capture more low-level interactions [6]. This result suggests that the dimensions of social anxiety and PSU are correlated because “afraid of negative evaluation” acts as a bridge symptom between these two networks. In other words, #SAS 2 (“afraid of negative evaluation”) serves a central role in the relationship between PSU and social anxiety. In line with this, a previous study also suggested that “fear of negative evaluation” is closely related to higher levels of PSU [49], which partially supports our findings. Research comparing traditional cognitive behavioral therapy (CBT) and mindfulness-based interventions, such as mindfulness-based stress reduction (MBSR), demonstrates comparable efficacy in addressing afraid of negative evaluation [50]. Thus, intervention selection can be tailored to individual needs, a strategy that may also extend to PSU.

### Gender differences in the networks of social anxiety and PSU symptoms

Despite demographic differences between men and women, sensitivity analysis revealed that these variables did not influence the results of the current study. The network structure varied by gender, and some symptoms of interaction were unique to boys or girls. The maximum edge strength [i.e., #MPAI 2 (“feeling anxious and lost”) and #MPAI 4 (“productivity loss”)] was greater in boys than in girls. Gender may moderate the relationship between social anxiety and PSU [51]. Furthermore, the NCT showed that the boys had a significantly greater connection between #SAS 2 (“afraid of negative evaluation”) and #SAS 3 (“anxiety in normal situation”) than the girls. The gender differences in social anxiety disorders are consistent with the findings of previous studies using NCT analysis [52]. A previous study showed that men who suffer from social anxiety may be more prone to self/identity-related distress than women [53]. Boys are more vulnerable than girls due to their tighter network structure.

Interestingly, in contrast to the network structure, our results showed that girls reported significantly higher scores than boys in all three dimensions of social anxiety. Females tend to report higher levels of body dissatisfaction than males [54], which may lead to higher levels of social body anxiety [55]. In addition, the strong interrelationship among symptoms within the network facilitates both the onset and persistence of the disorder [23]. We posit that this robust association does not signify a more severe disorder; rather, it serves as an indicator of comparable sensitivity. Individuals with social anxiety often perceive themselves as lacking in instrumentality [56], such as independence (e.g., competitiveness), are typically associated with traditional masculinity [57]. Moreover, cultural differences in gender role identification are associated with social anxiety, with Asian Canadians more vulnerable due to weaker masculine identification, while women report higher social anxiety across cultures [58]. In Chinese culture, men report greater engagement in conflict-reducing behaviors and lower aggression compared to Western culture [59]. Therefore, masculinity may mitigate social anxiety in boys, but cultural factors, particularly in Asian contexts, can heighten vulnerability due to lower masculinity. This result may explain the inconsistent findings of gender differences in previous studies [12, 25, 53].

The most important finding is that within the social anxiety and PSU symptomatology network in boys, there were significantly greater edge weights between PSU and social anxiety [i.e., #MPAI 2 (feeling anxious and lost)—#SAS 2 (“afraid of negative evaluation”)] in boys than in girls. The centrality results suggest that #MPAI 2 (“feeling anxious and lost”) and #SAS 3 (“anxiety in normal situation”) are more prominent for boys than girls. The motivations for smartphone addiction differ between men and women, with women being more sensitive to internal motivations and men being more sensitive to external motivations [60]. A previous study showed that there are gender differences in the specific methods of smartphone use [61]. Male adolescents who cannot use smartphones to play games may experience anxiety and loss [62]. With the rapid development and popularity of mobile video games, males have reported spending significantly more time playing mobile video games than females [63]. They may view video games as a way to escape the frustrations of reality, which in the long run may lead to increased negative feelings such as anxiety and a sense of loss. When individuals spend too much time on smartphones to reduce their negative feelings, they may become more afraid of negative evaluation. In contrast to boys, female adolescents prefer to use smartphones for communication functions [60, 62]. These functions may reduce social anxiety to some extent. The results of these gender differences in network structure may explain the inconsistencies of previous findings [26, 32].

However, centrality invariance tests show that the #MPAI 1 (“inability to control craving”) is more influential and central for girls. Previous studies suggest that girls have significantly higher levels of PSU and suffer more from the negative impact of PSU than boys [64, 65]. Adolescent females exhibit significantly higher levels of smartphone dependence and influence than males [64], characterized by an “inability to control craving,” which is strongly associated with negative outcomes, including depression [65].

In summary, smartphone use patterns and motivations may clarify gender differences. In addition, these findings indicate that tailored interventions addressing gender-specific mobile phone use patterns may more effectively mitigate symptoms of PSU and social anxiety among adolescents. Certain interventions can be implemented by parents independently, whereas others may necessitate the involvement of mental health professionals. For girls, interventions aimed at mitigating craving control difficulties, such as limiting exposure to mobile phone stimuli [66], should complement traditional CBT or MBSR. Addressing boys' emotional states is vital in the context of social anxiety and PSU, with targeted interventions such as distraction, reappraisal, and venting shown to be more effective for boys than for girls [67]. Moreover, the findings of this study have implications for educators, and policymakers. For example, educators should prioritize strategies aimed at reducing the reliance on smartphones and minimizing the exposure to mobile devices, with particular attention to the potential impact on girls. Policymakers should consider implementing measures to regulate adolescent PSU and develop accessible intervention strategies (e.g., reducing academic burdens and promoting balanced academic expectations) aimed at mitigating the negative emotional impacts on students, with particular focus on boys.

## Limitations and future study

Several limitations of the current study need to be mentioned. First, the data were self-reported by adolescents, and the objectivity and authenticity may have been undermined by subjectivity. The sample's diversity is critical; this study focused solely on a Chinese adolescent population and was regionally concentrated, which raises concerns about the generalizability of the findings, particularly to Western populations (e.g., European or American). Future research should incorporate multi-source data, such as parent-reported, biological, and behavioral (e.g., objective smartphone usage logs) data, to mitigate social validation effects and subjective bias.

Additional gender-related factors, including constructs such as masculinity or cultural orientation, should also be taken into account. Second, although this study focused on the interactions of social anxiety and PSU, there are still many comorbidities associated with PSU that may occur in individuals with different mental health conditions, including depression, anxiety, and self-injury [68, 69]. Hence, subsequent research should take into account additional potential confounding variables (i.e., socioeconomic status, academic pressures, or parental involvement) associated with PSU. Third, although this study identified the interactions and gender differences in the relationship between social anxiety and PSU based on a large sample, experimental or longitudinal studies are still needed to explore the effectiveness of interventions targeting comorbidity or gender differences and the underlying mechanisms involved. Undirected networks may complicate clinical interventions, as causality cannot be established, and targeted symptoms may be influenced by other symptoms rather than causing them. The current network model focuses on interindividual analysis and neglects individual heterogeneity, resulting in symptom relationships that may not be universally applicable.

In summary, these limitations affect the interpretation of the findings by potentially compromising result generalizability, overlooking the role of other mental disorders, limiting causal inferences due to the absence of experimental data.

## Conclusions and implications

The present study explored the relationship between social anxiety and PSU through a network analysis approach. The results suggest that “productivity loss” and “afraid of negative evaluation” serve as the most influential symptoms among all adolescents, and the edges between “anxiety in unfamiliar situation” and “afraid of negative evaluation” and between “anxiety in normal situation” and “afraid of negative evaluation” were strong among all adolescents. Additionally, “afraid of negative evaluation” was identified as the bridging factor. For practical implications, future interventions could incorporate self-guided virtual reality exposure therapy [70], which may lead to lasting improvements in how socially anxious individuals perceive the threat of negative evaluation from others [71]. This approach could help them develop the ability to remain unafraid of negative comments, thereby reducing the interaction between PSU use and social anxiety. Moreover, the current study showed that females scored higher on all three dimensions of social anxiety than males. Although there was no significant gender difference in global strength, the expected influence for “inability to control craving”, “feeling anxious and lost”, and “anxiety in normal situation” were significantly different between the genders.

## Supplementary Information

Supplementary Material 1.

## Data Availability

No datasets were generated or analysed during the current study.

## Abbreviations

PSU: Problematic smartphone use
CIUT: Compensatory internet use theory
MPAI: Mobile phone addiction index
SAS: Social anxiety scale
EBIC: Extended Bayesian information criterion
LASSO: Least absolute shrinkage and selection operator
*EI*: Expected influence
*CS-C*: Correlation stability coefficient
NCT: *NetworkComparisonTest*

## Code availability

Analytic code and data for this work are available upon request.
