# Supplementary material for: Gender differences in symptom interactions between problematic smartphone use and social anxiety in adolescents: a network analysis
Source: Child Adolesc Psychiatry Ment Health. 2025 Feb 14;19:9. doi: 10.1186/s13034-025-00865-w (PMC11829345; doi:10.1186/s13034-025-00865-w)
Supplement: Supplementary file 1 — Supplementary Material 1. [file 13034_2025_865_MOESM1_ESM.docx]

**Gender differences in symptom interactions between problematic smartphone use and social anxiety in adolescents: A network** **analysis**

***Supplemental Information***

**Contents**

[Supplemental Tables 2](#_Toc188296633)

[Table S1: Demographic characteristics (*N* = 2918). 2](#_Toc188296634)

[Table S2: The Weight-Matrix, Predictability, Bridge EI, and Centrality of the Network. 4](#_Toc188296635)

[Supplementary Figures 6](#_Toc188296636)

[Fig.S1. Bootstrapped stability test (α=0.05) for edge-weight. 6](#_Toc188296637)

[Fig.S2. Bootstrapped stability test (α=0.05) for expected influence (EI) and bridge EI of nodes. 7](#_Toc188296638)

[Fig.S3. Nonparametric bootstrapped confidence intervals of estimated edges. 8](#_Toc188296639)

# Supplemental Tables

## Table S1: Demographic characteristics (*N* = 2918).

| **Demographic characteristics** | ***M (SD) or N* (%)** | | **Gender differences** | | |  |
| --- | --- | --- | --- | --- | --- | --- |
|  |  |  | **Males** | **Females** | ***p*** ^(a)^ | |
| ***N*** | 2918 |  | 1380 | 1538 | **< .01** | ** |
| **Age** | 14.73 | (1.39) | 14.77 (1.35) | 14.69 (1.43) | .138 |  |
| **Education** |  |  |  |  |  |  |
| Junior high school | 2369 | (81.19%) | 1098 | 1271 | **< .05** | * |
| Senior high school | 549 | (18.81%) | 282 | 267 |  |  |
| **School location** |  |  |  |  |  |  |
| City | 1185 | (40.61%) | 567 | 618 | .646 |  |
| County | 1733 | (59.39%) | 813 | 920 |  |  |
| **Stay at Home Kids (Y)** | 86 | (2.95%) | 37 | 49 | .196 |  |
| **Only child (N)** | 1980 | (67.85%) | 900 | 1080 | **< .001** | *** |
| **Health problem** |  |  |  |  |  |  |
| Critical physical illness | 12 | (0.41%) | 8 | 4 | .287 |  |
| Mental illness | 19 | (0.65%) | 3 | 16 |  |  |
| None | 2887 | (98.94%) | 1369 | 1518 |  |  |
| **Parental relationships** |  |  |  |  |  |  |
| Harmonious relationship, never argue | 1365 | (46.78%) | 695 | 670 | **< .001** | *** |
| Occasional arguments, 1-2 times per month | 1371 | (46.98%) | 614 | 757 |  |  |
| Frequent arguments, almost half of the days each month | 129 | (4.42%) | 54 | 75 |  |  |
| Constant arguments, almost every day | 53 | (1.82%) | 17 | 36 |  |  |
| **Parental marriage (divorced)** | 189 | (6.48%) | 81 | 108 | **< .05** | * |
| **If your parents divorced, who you lived with now?** |  |  |  |  |  |  |
| Mother | 107 | (56.61%) | 38 | 69 | **< .01** | ** |
| Father | 60 | (31.75%) | 36 | 24 |  |  |
| Relatives of the father or mother | 22 | (11.64%) | 7 | 15 |  |  |

*Note.* (a) T test or Chi-square tests were used. * *p* < .05, ** *p* < .01, *** *p* < .001

## Table S2: The Weight-Matrix, Predictability, Bridge EI, and Centrality of the Network.

|  |  |  | **Weight-Matrix** | | | | | | | **Predictability** | **Bridge EI** | **EI** |
| --- | --- | --- | --- | --- | --- | --- | --- | --- | --- | --- | --- | --- |
| **Network** |  | **Node (Dimension)** | **MPAI 1** | **MPAI 2** | **MPAI 3** | **MPAI 4** | **SAS 1** | **SAS 2** | **SAS 3** |  |  |  |
| **Full samples** |  | **MPAI** |  |  |  |  |  |  |  |  |  |  |
|  |  | MPAI 1 | 0 | 0.339 | 0.022 | 0.436 | 0.039 | 0.042 | 0.011 | 0.67 | -0.11 | -0.35 |
|  |  | MPAI 2 | 0.339 | 0 | 0.321 | 0.169 | -0.073 | 0 | 0.134 | 0.65 | -1.01 | -0.30 |
|  |  | MPAI 3 | 0.022 | 0.321 | 0 | 0.363 | 0.024 | 0.075 | -0.012 | 0.61 | -0.26 | -1.19 |
|  |  | MPAI 4 | 0.436 | 0.169 | 0.363 | 0 | 0.069 | 0.043 | -0.017 | 0.71 | 0.00 | 1.38 |
|  |  | **SAS** |  |  |  |  |  |  |  |  |  |  |
|  |  | SAS 1 | 0.039 | -0.073 | 0.024 | 0.069 | 0 | 0.412 | 0.351 | 0.69 | -1.07 | -0.95 |
|  |  | SAS 2 | 0.042 | 0 | 0.075 | 0.043 | 0.412 | 0 | 0.476 | 0.74 | 1.85 | 1.25 |
|  |  | SAS 3 | 0.011 | 0.134 | -0.012 | -0.017 | 0.351 | 0.476 | 0 | 0.72 | 0.58 | 0.16 |
| **Males** |  | **MPAI** |  |  |  |  |  |  |  |  |  |  |
|  |  | MPAI 1 | 0 | 0.304 | 0.026 | 0.415 | 0.056 | 0.028 | 0 | 0.64 | -0.26 | -0.71 |
|  |  | MPAI 2 | 0.304 | 0 | 0.308 | 0.234 | -0.058 | 0.042 | 0.121 | 0.68 | 0.43 | 0.25 |
|  |  | MPAI 3 | 0.026 | 0.308 | 0 | 0.359 | 0.014 | 0.067 | 0 | 0.61 | -0.36 | -1.22 |
|  |  | MPAI 4 | 0.415 | 0.234 | 0.359 | 0 | 0.052 | 0 | 0 | 0.71 | -1.31 | 1.12 |
|  |  | **SAS** |  |  |  |  |  |  |  |  |  |  |
|  |  | SAS 1 | 0.056 | -0.058 | 0.014 | 0.052 | 0 | 0.381 | 0.350 | 0.70 | -0.92 | -1.09 |
|  |  | SAS 2 | 0.028 | 0.042 | 0.067 | 0 | 0.381 | 0 | 0.528 | 0.77 | 1.47 | 1.09 |
|  |  | SAS 3 | 0 | 0.121 | 0 | 0 | 0.350 | 0.528 | 0 | 0.76 | 0.95 | 0.56 |
| **Females** |  | **MPAI** |  |  |  |  |  |  |  |  |  |  |
|  |  | MPAI 1 | 0 | 0.365 | 0.025 | 0.457 | 0.028 | 0.051 | 0.030 | 0.69 | 0.17 | 0.18 |
|  |  | MPAI 2 | 0.365 | 0 | 0.327 | 0.114 | -0.074 | 0 | 0.104 | 0.64 | -1.40 | -0.78 |
|  |  | MPAI 3 | 0.025 | 0.327 | 0 | 0.363 | 0.028 | 0.059 | 0 | 0.61 | -0.27 | -1.04 |
|  |  | MPAI 4 | 0.457 | 0.114 | 0.363 | 0 | 0.075 | 0.076 | -0.025 | 0.72 | 0.51 | 1.39 |
|  |  | **SAS** |  |  |  |  |  |  |  |  |  |  |
|  |  | SAS 1 | 0.028 | -0.074 | 0.028 | 0.075 | 0 | 0.423 | 0.350 | 0.68 | -0.87 | -0.81 |
|  |  | SAS 2 | 0.051 | 0 | 0.059 | 0.076 | 0.423 | 0 | 0.441 | 0.73 | 1.70 | 1.28 |
|  |  | SAS 3 | 0.030 | 0.104 | 0 | -0.025 | 0.350 | 0.441 | 0 | 0.69 | 0.17 | -0.22 |

***Note*. EI, expected Influence.**

# Supplementary Figures


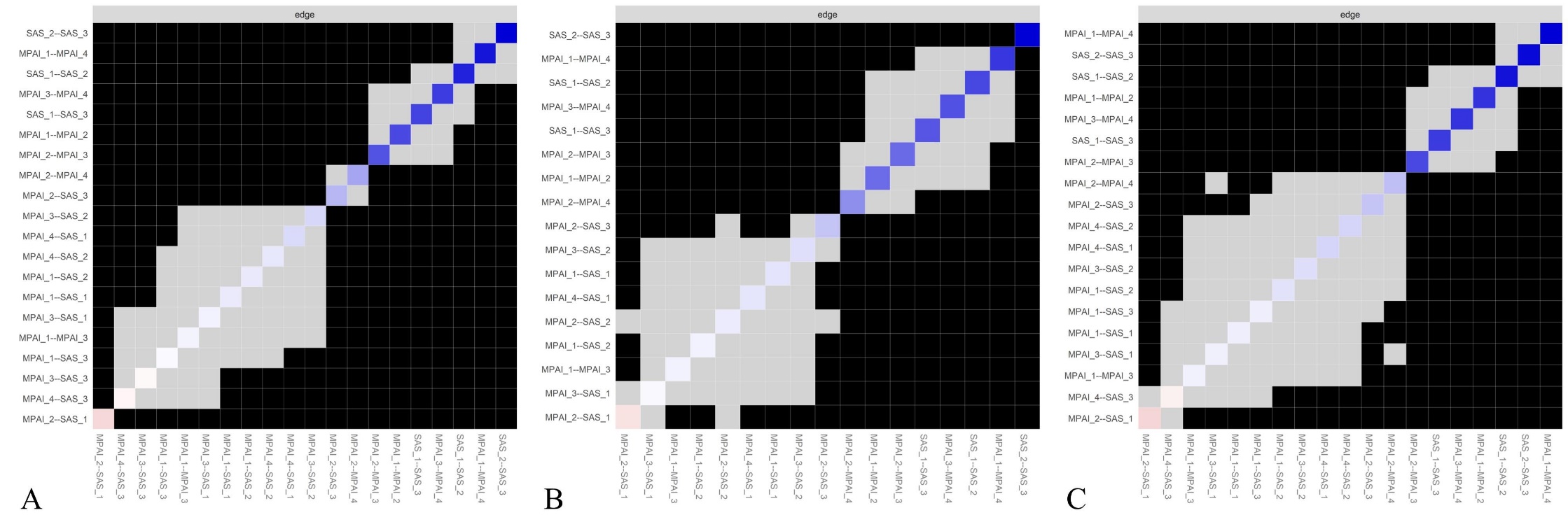


Fig.S1. Bootstrapped stability test (α=0.05) for edge-weight. The color of the boxes indicates whether edge-weights differ significantly from each other (i.e., black) or do not differ significantly (i.e., grey). The diagonal line represents the strength of edge weights, ranging from red (indicating negative associations) to white (representing weaker edges) and finally blue (representing stronger edge weights). A, Full samples. B, males. C, females.


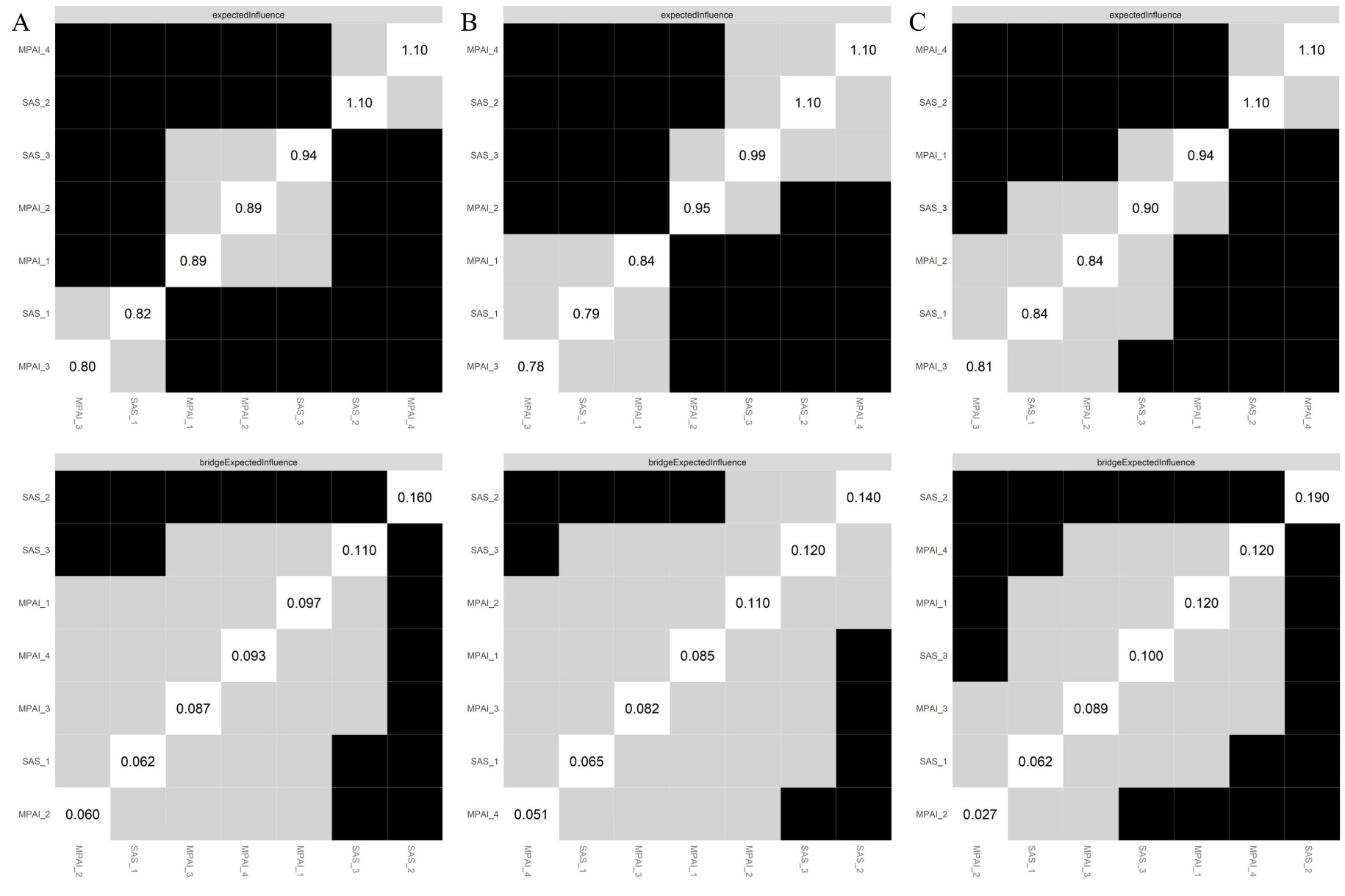


Fig.S2. Bootstrapped stability test (α=0.05) for expected influence (EI) and bridge EI of nodes. The color of the boxes represents whether the nodes differ significantly from each other (i.e., black) or do not differ significantly (i.e., grey). A, Full samples. B, males. C, females.

**
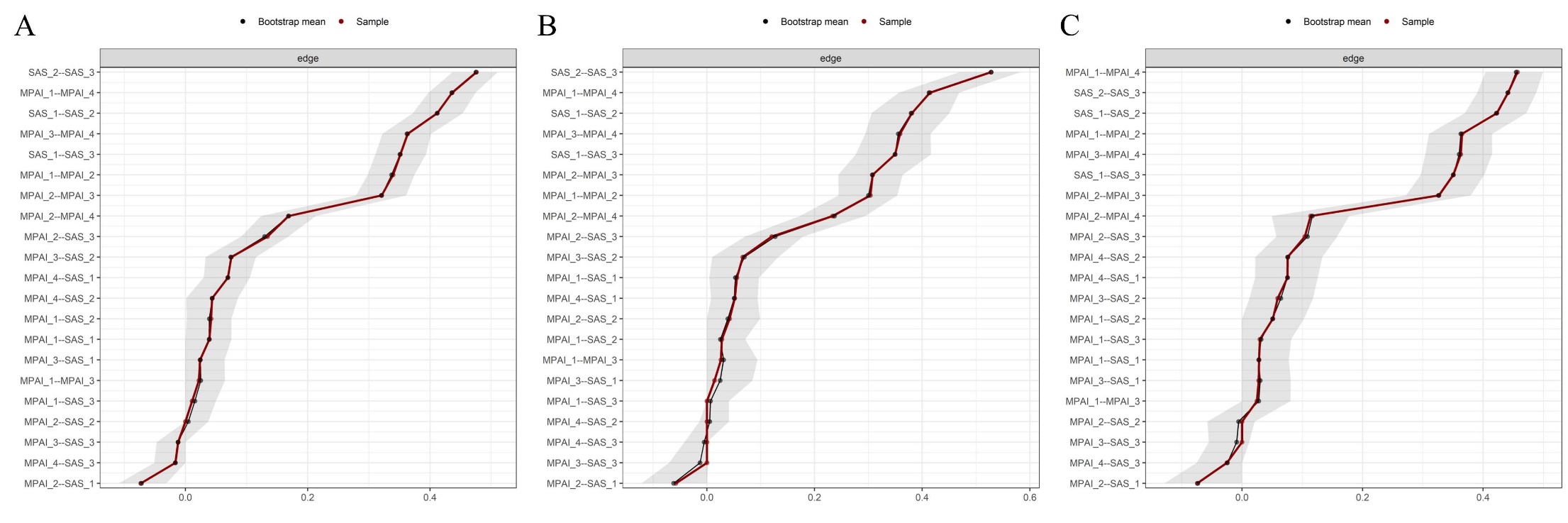
**

Fig.S3. Nonparametric bootstrapped confidence intervals of estimated edges. The red line represents the estimated edge, while the dark area indicates the 95% bootstrap confidence interval. A, Full samples. B, males. C, females.
